# Examining the Persuasive Effects of Health Communication in Short Videos: Systematic Review

**DOI:** 10.2196/48508

**Published:** 2023-10-13

**Authors:** Zicheng Zhu, Shiyu Liu, Renwen Zhang

**Affiliations:** 1 Department of Communications and New Media, Faculty of Arts & Social Sciences National University of Singapore Singapore Singapore; 2 Global Health Institute Xi'an Jiaotong University Xian China

**Keywords:** short video, persuasion, health, systematic review, mobile phone

## Abstract

**Background:**

The ubiquity of short videos has demonstrated vast potential for health communication. An expansion of research has examined the persuasive effect of health communication in short videos, yet a synthesis of the research is lacking.

**Objective:**

This paper aims to provide an overview of the literature by examining the persuasive effect of health communication in short videos, offering guidance for researchers and practitioners. In particular, it seeks to address 4 key research questions: What are the characteristics of short videos, samples, and research designs in short video–based health communication literature? What theories underpin the short video–based health communication literature? What are the persuasive effects of health communication in short videos? and What directions should future research in this area take?

**Methods:**

Following the PRISMA (Preferred Reporting Items for Systematic Reviews and Meta-Analyses) guidelines, an electronic search of 10 databases up to March 10, 2023, generated 4118 results. After the full-text screening, 18 articles met the eligibility criteria.

**Results:**

The current research lacks a uniform definition of short videos, demonstrates sample biases in location and education, and adopts limited methodologies. Most studies in this synthesis are theoretically grounded or use theoretical concepts, which are predominantly well examined in persuasion research. Moreover, relevant topics and suitable themes are effective in persuasive health communication outcomes, whereas the impact of diverse narrative techniques remains ambiguous.

**Conclusions:**

We recommend that future research extends the definition of short videos beyond time constraints and explores non-Western and less-educated populations. In addition, researchers should consider diverse methods to provide a more comprehensive examination and investigate the impact of audience targeting and narrative techniques in short video health communication. Finally, investigating how the unique aspects of short videos interact with or challenge traditional persuasion theories is essential.

## Introduction

### Background

Health communication scholars have used diverse modalities such as text, audio, and video to communicate health information to the public [1]. Many studies have demonstrated that compared with other modalities, video is more effective in disseminating health information owing to its engrossing nature [2], visual appeal, and ability to illustrate complex concepts [3]. According to the dual coding theory, people process information through 2 systems, 1 for visual information and another for verbal information [4]. The use of visual and verbal information in videos allows both systems to process and store information. Health messages disseminated through videos are more memorable because viewers can retrieve both imagery and verbal information.

In recent years, short videos have gained significant popularity and have become influential in people’s lives. TikTok, the most popular short video app, was downloaded globally more times than any other app in 2021 [5]. Instagram Reels, a short video feature on Instagram, has 2.35 billion monthly active users [6]. Similarly, YouTube Shorts has seen a remarkable 135% growth year over year, now boasting >1.5 billion monthly users [7]. Moreover, 85% of the marketers have identified short videos as the most effective format on social media [8]. The appeal of short videos is attributed to their ease of production, including easy editing of music, animation, and visual effects; convenient distribution; and their ability to cover a broad spectrum of topics, such as beauty, cooking, education, and technology [9].

Among various topics on short video platforms, health-related topics have grown extensively. During the COVID-19 pandemic, the World Health Organization used TikTok to share reliable information and counter misinformation regarding the virus [10]. As of July 12, 2020, videos with COVID-19–related hashtags on TikTok had received a total of 130.8 billion views [11]. The affordances of short video platforms, including live streaming, searching, metavoicing, and recommending, incentivize people to seek health information on the platforms [12]. Therefore, short videos have become a powerful tool for providing health advice and influencing people’s health beliefs and behaviors [13]. Consequently, understanding how to disseminate health information through short videos is crucial.

Thus far, a large body of work has examined the spread and impact of health-related information on short video platforms. For example, Basch et al [14] and Yalamanchili et al [15] examined COVID-19–related and vaccination-related content on TikTok and found that videos featuring humor, music, and dance were positively related to user engagement, as measured through views, comments, and likes. Understanding the factors that contribute to user engagement is essential. However, health communication researchers are also interested in the factors that influence the effects of short videos on users’ health attitudes and behaviors, which may inform the design of effective health campaigns. Therefore, an increasing number of studies have examined the persuasive effects of different designs of health-related short videos. Lipkus and Sanders [16], for instance, evaluated the effects of short videos with various themes of waterpipe tobacco smoking (WTS) on young adults’ risk beliefs and attitudes toward WTS.

Despite this, systematic efforts to analyze the persuasive effects of health communication through short videos remain scarce, leading to gaps in our understanding of this phenomenon. A recent systematic review by McCashin and Murphy [17] investigated the use of TikTok for public health and mental health purposes and concluded that the platform holds promise as a means of disseminating health information to young audiences. Nonetheless, most of the studies within the review by McCashin and Murphy [17] focused on the relationship between TikTok health video features and user engagement, neglecting to explore persuasive outcomes. Previous research has also examined the impact of video-based education on behavioral modification, revealing that effectiveness is contingent upon target behaviors and that gain-framed messages are more efficacious [18]. However, the average video length in these studies was 36 minutes, which exceeded the typical duration of short videos, rendering these findings inadequate for understanding the impact of health communication through short videos.

### Objective

To address these knowledge gaps, this paper synthesizes the existing literature on the persuasive effects of health communication in short videos. Central to this review is a focus on studies that explore various design strategies of health-related short videos, rather than those comparing the persuasive efficacy of short videos with no videos or other media modalities, such as text or images. This focus of our study emerged from the ubiquity of short videos in today’s digital landscape, which shifts the main inquiry of stakeholders from whether to use short videos for health communication, to determine the best design strategies for maximum impact. Although studies contrasting short videos with no videos or other mediums offer insights into the overall viability of short videos for health communication, research into specific design elements directly addresses the main inquiry: discerning the most effective design strategies for health persuasion in short videos. Specifically, health communication in this paper is defined as the design and dissemination of health-related messages to influence individual and community decisions promoting health [19]. Given the lack of a consensus on short video duration, we refrained from imposing a specific time constraint. Instead, we focused on studies that self-identify as examining short videos. Our study addressed 4 research questions (RQ):

- RQ1: What are the characteristics of short videos, samples, and research designs in short video–based health communication literature?
- RQ2: What theories underpin the short video–based health communication literature?
- RQ3: What are the persuasive effects of health communication in short videos?
- RQ4: What directions should future research in this area take?

The insights provided by this review hold significant value for health researchers, practitioners, and policy makers as they offer guidance for designing effective health messages in short videos and inform future investigations into the role of short videos in disseminating health information.

## Methods

### Data Sources and Search Strategy

Following the PRISMA (Preferred Reporting Items for Systematic Reviews and Meta-Analyses) model [20], we executed a systematic search across 8 primary databases: ACM Digital Library, PsycINFO, Web of Science, Communication & Mass Media Complete, MEDLINE, Embase, CINAHL, and Scopus. Google Scholar was used as a supplementary source to augment the initial search. In addition, we incorporated the ProQuest Dissertations & Theses Global database to identify doctoral dissertations, as the inclusion of gray literature in systematic reviews is considered crucial for mitigating publication bias [21]. The PRISMA checklist can be found at Multimedia Appendix 1.

As our review focuses on 3 main aspects, that is, short videos, health, and persuasive effects, we developed 3 sets of search strings (Textbox 1). Specifically, for short videos, we used the term *short video* along with other prominent short video platforms [22]. For health and persuasive effects, we drew from the studies by Ehsan et al [23] and Walter et al [24] to generate the search strings. Consequently, the search strings were formulated as follows: (“short video” OR tiktok OR douyin OR “youtube shorts” OR “instagram reels” OR triller OR “snapchat spotlight” OR vine OR “facebook shorts”) AND (persua* OR impact OR effect OR outcome OR belief OR attitude OR behavior OR behaviour OR intention OR knowledge) AND (health OR medic* OR clinical OR disease OR disabilit* OR disorder OR ill* OR well-being OR wellbeing). We searched for these strings in the titles, abstracts, or keywords of articles within the databases. The search encompassed publications available up until the search date, March 10, 2023. The search strategies for all searched databases can be found in Multimedia Appendix 2.

**Search strings.**

**Short video**

- short video
- tiktok
- douyin
- youtube shorts
- instagram reels
- triller
- snapchat spotlight
- vine
- facebook shorts

**Health**

- health
- medic
- clinical
- disease
- disabilit
- disorder
- ill
- well-being
- wellbeing

**Persuasive effects**

- persua
- impact
- effect
- outcome
- belief
- attitude
- behavior
- behaviour
- intention
- knowledge

### Inclusion and Exclusion Criteria

Articles were included in our corpus if they (1) pertained to health; (2) investigated the content or characteristics of short videos; (3) related to persuasion, defined as a deliberate attempt to influence people’s attitudes, beliefs, intentions, or behaviors [25]; and (4) were empirical studies. We excluded articles that (1) were not health related, (2) did not explore the content or characteristics of short videos (eg, solely examined the impact of providing a short video vs no video on persuasive outcomes [26]), (3) were not pertinent to persuasion (eg, exclusively investigated user engagement with short videos [14]), (4) were not written in English, and (5) were not full articles. We did not exclude papers based on their methods or participants, as we aimed for inclusivity in our review.

### Study Screening Method

**Figure 1:**
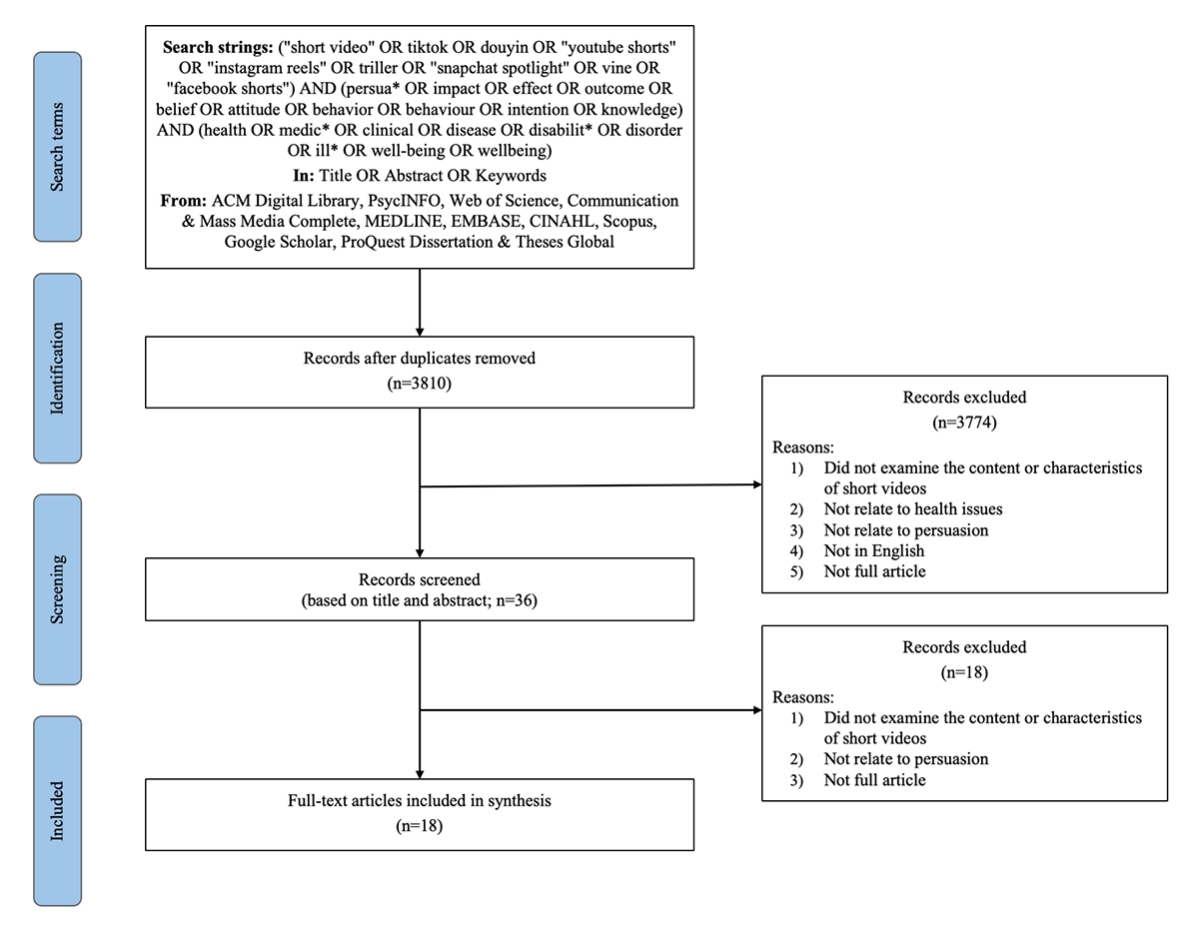
PRISMA (Preferred Reporting Items for Systematic Reviews and Meta-Analyses) diagram showing the flow of studies.

Following the initial search, we identified 4118 articles. After duplicate removal, 3810 articles remained. Adhering to the inclusion and exclusion criteria, 2 authors of this study jointly screened 9.97% (380/3810) of articles, yielding satisfactory intercoder reliability with Krippendorff α=.82. The remaining articles (n=3430) were evenly allocated between the 2 coders for further scrutiny. The results were discussed among all authors to ensure compliance with the inclusion and exclusion criteria. Ultimately, the final data set comprised 18 articles. We consulted scholars in the field to verify that no relevant literature was missing from the data set. Figure 1 illustrates the PRISMA flow diagram representing the search and screening process. The lists of papers examined at each stage are presented in Multimedia Appendices 3 to 6 [16,27-43].

### Data Extraction and Synthesis

The first author thoroughly reviewed the final articles and, in collaboration with the other authors, established a codebook. The coding decisions were informed by previous reviews on TikTok and health [17] and the RQs of this study. Specifically, we coded the study identification information, including authors, publication years, and study locations. In addition, we coded basic information related to the studies’ topics, such as the health domain and study objectives. To address RQ1, we coded the video length, sample size, age, race, sex, and education level of the sample; data collection method; and study duration. For RQ2, we coded the theories used in these studies. To answer RQ3, we coded the health message design, outcome variables, persuasive effects, moderators, mediators, and moderation and mediation effects. The first author coded all studies in the final data set and discussed the coding results with all authors to ensure accurate validation against the original papers. Table 1 presents an overview of the paper’s study characteristics, and Table 2 summarizes the persuasive effects. The complete coding is provided in Multimedia Appendix 7.

**Table 1:** Study characteristics of the papers.

Study, year	Health domain	Location	Video length	Sample	Data collection
de Groot et al [27], 2022	Albinism-related stigma	Tanzania	10 min 46 s; 9 min 14 s	Mean age 16.35 y; 52.1% male; 28.65% parents with higher education	Experiment; focus group
Jensen et al [28], 2022	COVID-19 vaccine	America	29-32 s	Mean age 40.7 (SD 11.2) y; 49.4% male; 73.4% White; 50.1% with a college degree	Experiment
Piltch-Loeb et al [29], 2022	COVID-19 vaccine	America	30 s	Mean age 40.8 (SD 11.8) y; 50% female; 74% White; 60% with a bachelor’s degree or higher	Experiment
Ruggs and McGonagle [30], 2022	Bias toward individuals with disabilities in the workplace	America	5 min; 6 min; 7 min 30 s	Study 1: mean age 40.59 (SD 10.11) y; 60.4% male; 83.2% White; 72.8% college degree; study 2: mean age 28.92 (SD 7.65) y; 79.4% female; 79% White; 75.1% with a college degree	Experiment
Xiao and Yu [31], 2022	Social distancing	China	N/A^a^	Mean age 27.27 (SD 5.8) y; 48% male	Experiment
Hood et al [32], 2022	Condom use	America	N/A	Mean age 19.38 (SD 14.19) y; 100% female; African American or Black; university students	Experiment
Jiang [33], 2021	Influenza vaccination	Hong Kong	5 min 1 s; 5 min 6 s	Mean age 34.78 (SD 14.19) y; 48.6% male; 37.3% bachelor’s degree	Experiment
Lipkus and Sanders [16], 2021	Waterpipe tobacco smoking	N/A	3 min 56 s; 4 min 33 s; 3 min 22 s; 2 min 44 s	Mean age 27.7 (SD 4.5) y; 46% male; 76% White; 88% with some college degree	Experiment
Wijayatunga et al [34], 2021	Weight bias	N/A	20 min	Intervention: mean age 37.79 (SD 13.4) y; 98% female; 85% White; registered dietitians; control 1: mean age 35.19 (SD 11.26) y; 98% female; 85% White; registered dietitians; control 2: mean age 36.85 (SD 10.48) y; 94% female; 83 White registered dietitians	Experiment
Baumeister et al [35], 2020	Blended therapy	German	5 min; 4 min	Mean age 48.6 (SD 11.7) y; 59% female; psychotherapists; race: N/A	Experiment
Ewing et al [36], 2020	Fear	United Kingdom	10 min	Mean age 40 y; majority White British or White other; education: N/A	Experiment
Violeau et al [37], 2020	Stigma of schizophrenia	France	1 min 40 s; 2 min; 1 min 40 s	Mean age 26 (SD 10.4) y; 34% male	Experiment
Davis et al [38], 2019	Glaucoma eye drop technique	America	4 min	Mean age 69.2 (SD 10.7) y; 51% male; 59% non-Hispanic White; 15-16 y of education	Experiment
Yoh et al [39], 2019	Safe driving	Taiwan	4 min	45% mean age 29 y; 32% mean age 30-39 y; 66% female	Survey
González-Cabrera and Igartua [40], 2018	Pregnancy prevention	Spain	8 min 41 s; 8 min 41 s	Mean age 15-16 (SD 0.88) y; 100% female; education: student	Experiment
Freeburn [41], 2017	Help-seeking	Canada	2-3 min	N/A	Experiment
Bearth et al [42], 2016	Food additives	Switzerland	3 min	Mean age 54 (SD 17) y; 52% male	Experiment
Whittaker et al [43], 2011	Smoking cessation	New Zealand	25 s	Mean age 27 (SD 8.7) y; 52.7% female; majority New Zealand European; education: N/A	Experiment

^a^N/A: not applicable.

**Table 2:** Persuasive effects and theories examined in the papers.

Study, year	Theories	Design of health messages	Persuasive effects
de Groot et al [27], 2022	N/A^a^	The themes of the topics (contact vs education)	Education group scored higher in albinism knowledge; no significant difference in attitude changes and knowledge sum scale.
Jensen et al [28], 2022	Theory of planned behavior	The relevance of the topics (related to content vs placebo) The themes of the topics (attitudes about vaccine safety; normative beliefs about the subjective, social norm to get vaccinated; response efficacy; self-efficacy)	Increased vaccination intentions for all treatment messages; treatment safety and response efficacy messages more effective in arousing self-efficacy and response efficacy, separately; no systematic effects on subjective norms, safety concerns, or protecting others.
Piltch-Loeb et al [29], 2022	Inoculation theory	The relevance of the topics (no vaccine-related content vs vaccine-related content)	Inoculated participants showed better misinformation recognition, less false information sharing, and higher vaccine willingness.
Ruggs and McGonagle [30], 2022	Model of factors affecting the treatment of disabled individuals in organizations	The themes of the topics (education vs socialization vs combination of education and socialization vs a control video about workplace diversity broadly)	Positive changes in knowledge and awareness for education and combined videos, and cognitive attitudes for socialization video; improvement in hiring scenario rating for education video.
Xiao and Yu [31], 2022	N/A	The narrative techniques (humorous vs rational)	Humorous message reduced risk perception compared with rational message.
Hood et al [32], 2021	N/A	The themes of the topics (affective vs cognitive)	Affective message led to more positive attitudes and intentions, and higher interest in receiving free condoms than cognitive message.
Jiang [33], 2021	N/A	The narrative techniques (narrative vs informational)	Narrative condition produced greater perceived threat; no significant difference in perceived efficacy, vaccination intent, or uptake.
Lipkus and Sanders [16], 2021	Theory of planned behavior	The themes of the topics (harms of WTS^b^, WTS myths, harms to others, addiction)	More risk perception of WTS with physical harms and harms to others themes; physical harm more effective at reducing susceptibility; no variation in average risk beliefs by video theme.
Wijayatunga et al [34], 2021	Attribution theory	The relevance of the topics (related to obesity vs not related to obesity) The relevance of the topics (uncontrollable causes vs controllable causes)	No significant difference among groups (*P*=.77, *P*=.75, *P*=.73, and *P*=.24).
Baumeister et al [35], 2020	Unified theory of acceptance and use of technology	The relevance of the topics (example of blended therapy vs placebo video)	Intervention group showed higher acceptance, performance expectancy, effort expectancy, and facilitating conditions for blended therapy; no significant difference in social influence (*P*=.30) or internet anxiety (*P*=.75).
Ewing et al [36], 2020	N/A	The relevance of the topics (managing fear vs learning to read)	Improved approach parenting and child steps toward feared stimulus for parenting tutorial group.
Violeau et al [37], 2020	N/A	The relevance of the topics (schizophrenia related content vs irrelevant content) The themes of the topics (activating continuum beliefs vs reducing continuum beliefs)	Continuum video reduced public stigma and increased self-stigma compared with neutral and categorial videos.
Davis et al [38], 2019	Social cognitive theory	The relevance of the topics (Meducation eye drop technique video vs nutrition video)	Better technique and higher self-efficacy in intervention group after the video and at 1 month.
Yoh et al [39], 2019	N/A	The video’s appeal (nice character, appropriate video length, and ease of understanding)	Video appeal improvements led to better educational effects and safety awareness.
González-Cabrera and Igartua [40], 2018	N/A	The narrative techniques (testimonial vs dialogic)	No significant difference among groups.
Freeburn [41], 2017	Theory of planned behavior	The relevance of the topics (mental health video vs control video)	No significant difference among groups (*P*=.21).
Bearth et al [42], 2016	N/A	The relevance of the topics (scientific risk assessment of food additive vs irrelevant topic)	Experimental group showed increased knowledge, positivity, acceptance, and reduced risk perception after the video.
Whittaker et al [43], 2011	Social cognitive theory	The relevance of the topics (role models discussing issues vs general health message)	No significant difference among groups (*P*=.70, *P*=.80, *P*=.80, *P*=.30, and *P*=.99).

^a^N/A: not applicable.

^b^WTS: waterpipe tobacco smoking.

## Results

Current research on health communication in short videos predominantly focuses on three themes: (1) the prevention of unhealthy behaviors, particularly among those susceptible to such actions (eg, WTS; 2/18, 11%); (2) diminishing health-related stigma (eg, albinism; 4/18, 22%); and (3) promoting the adoption or reduction of specific behaviors (eg, influenza vaccination; 12/18, 67%). In the following sections, we provide a more comprehensive account of the studies’ basic characteristics, the theories used, and the persuasive impact of health communication in short videos.

### Study Characteristics

The research methodology used in the examined studies exhibits limited variation and potential issues with sample representation, which may subsequently affect the generalizability of the findings. Of the 18 studies, 16 (89%) used experimental designs, whereas 1 (5%) study used a survey approach [39] and 1 (5%) study implemented mixed methods, incorporating a focus group after the experiment [27]. In addition, the samples in the investigated studies displayed a bias concerning location and education. Most studies (12/18, 67%) were conducted in Western countries (ie, Europe, North America, and Oceania), with only 4 studies undertaken in Africa and Asia. Two studies did not report the study location, but their participants were predominantly White [16,34]. Notably, such a location bias may arise from the review’s inclusion criteria, which only considered studies written in English. Consequently, research conducted in non-Western countries and published in languages other than English might have been overlooked. Among the studies targeting adults and presenting participants’ education statistics, most of the participants (9/18, 50%) held at least some college degrees. The generalizability of the persuasive effects identified in existing studies to other countries and individuals with lower educational levels remains uncertain.

In addition, current research lacks a precise definition of short videos in terms of video length. Although most studies (14/18, 78%) feature short videos that do not exceed 10 minutes in duration—the maximum video length for TikTok as of February 2022 [44]—1 study included short videos of approximately 20 minutes [34]. Furthermore, among studies with videos shorter than 10 minutes, only 3 studies had videos ≤1 minute, a typical video length for short video platforms [45].

### Theories

Most studies in this synthesis are either theoretically grounded (n=9) or used variables guided by theoretical concepts. However, the theories and theoretical concepts used in these studies are predominantly well examined, particularly in persuasion research. For instance, 6 studies used theories addressing factors influencing individuals’ behavioral intentions and behaviors—specifically, social cognitive theory, the theory of planned behavior, and the unified theory of acceptance and use of technology. The factors examined in these theories encompass the observation of others’ behaviors, norms and social influence, attitudes, behavioral control, and expectancy. These theories have been frequently applied to understand people’s acceptance of new technology and advocated behaviors [46].

In addition, 3 studies used theories concerning people’s beliefs and attitudes, namely inoculation theory, attribution theory, and a specific theoretical model addressing the treatment of disabilities in the workplace [29,30,34]. These theories aim to comprehend the impact of people’s thinking processes (ie, attribution process, the amount of information acquired, and pre-exposed counterargument) on their subsequent perceptions and reactions. Arguably, the application of these theories offers insights for designing health messages in short videos in the included studies. Nevertheless, few studies have investigated how the unique aspects of short videos extend or challenge these traditional theories and contribute to these theories.

### Persuasive Effects of Health Communication in Short Videos

#### Overview

The studies included in this review examined diverse designs of health messages in short videos, reporting a variety of health-related outcomes such as attitudes and beliefs (14/18, 78%), behavioral intentions (10/18, 56%), and health-related knowledge (6/18, 33%). Remarkably, only 4 studies within the synthesis assessed individuals’ health behaviors. Despite the significance of attitudes and intentions as antecedents of behavior [47], the potential for attitude-behavior discrepancies cannot be dismissed [48]. Consequently, the extent to which alterations in individuals’ attitudes and intentions may elicit changes in their behavior remains unclear. In addition, 72% (13/18) of the studies assessed outcomes immediately following interventions, whereas the remaining 28% (5/18) were longitudinal studies, measuring outcomes from 1 week to 6 months after the intervention. Furthermore, 67% (12/18) of the studies gauged participants’ pre-exposure attitudes, beliefs, or behaviors concerning central health issues. In the following sections, we discuss the primary effects of health message design in short videos, accompanied by an analysis of examined moderators and mediators.

#### Main Persuasive Effects of the Design of Health Messages in Short Videos

The included studies investigated various aspects of health message design in short videos, including topic relevance, topic theme, and narrative and video appeal strategies.

##### Topic Relevance

Topic relevance, investigated in 10 studies, refers to the effectiveness of presenting health-related topics compared with unrelated topics for persuasion. For example, Davis et al [38] assessed the effectiveness of a glaucoma eye drop technique video against a general nutrition video in improving patients’ technique. Studies of this kind produced mixed results; some found relevant topics more persuasive [28,35], whereas others observed nonsignificant differences [41,43]. However, factors such as suboptimal sampling recruitment, high attrition rates, and low character-audience relatability may contribute to these observed insignificances [41,43].

##### Topic Theme

Overall, 9 studies examined topic themes. Demonstrating that distinct themes significantly influence persuasive outcomes, these studies underscore the importance of selecting suitable themes for health communication in short videos. For example, physical harm, harm to others, and myth-related themes were more effective than addiction themes in modifying WTS perceptions [16]. Educational content was more effective than contact content, featuring individuals living with a disease in increasing disease-specific knowledge and enhancing the assessments of affected individuals [27,30]. Generally, themes addressing affective stimuli processing and response efficacy were more persuasive than their counterparts [28,32]. There is 1 exception indicating that varying attribution styles of a health issue (ie, controllable vs uncontrollable) did not significantly influence weight bias [34]; this outcome may be attributable to the study’s sample comprising registered dietitians who had previously participated in weight bias reduction training programs, potentially limiting further improvement in their weight bias levels. This underscores the significance of selecting an appropriate sample, as participants with preexisting elevated baseline perceptions, attitudes, and intentions toward the focal issue may exhibit minor changes in persuasive outcomes.

##### Narrative and Video Appeal Techniques

The third dimension, narrative and video appeal techniques, investigated the persuasive impact of varying strategies and appeals while maintaining consistent themes across different short videos. Video appeal demonstrated promising effects, as engaging, appealing, and comprehensible short videos were more likely to inspire behavioral intentions [39]. Conversely, the persuasive impact of narrative techniques remains ambiguous, with studies reporting inconclusive and mixed outcomes [40]. This may correspond with previous findings suggesting smaller effect sizes for videos compared with other modalities such as video games and text [49], which may be attributed to the fact that reading necessitates ongoing mental simulation and video games are intrinsically richer in sensory input, whereas processing visual stimuli involves a more automatic process requiring minimal mental investment [50].

In addition, research examining narrative techniques indicated that narrative content was more effective than informational content in altering individuals’ perceptions of influenza threat but not their perceived efficacy, intentions, or behavior [33]. This discrepancy may be ascribed to the videos’ theme emphasizing the interconnected nature of influenza rather than the efficacy aspects of vaccination, once again highlighting the importance of short video themes. Furthermore, this implies a potential interaction effect between the theme and narrative technique in short videos, where the influence of narrative techniques may be undermined without an appropriate theme. Xiao and Yu [31] found that humorous short video messages increased message likability but reduced individuals’ risk perception, emphasizing the need for additional inquiries into the intricate balance between attention-grabbing narrative methods and achieving the intended persuasive results.

#### Moderators and Mediators

In light of the inconclusive findings regarding the primary persuasive effects of health messages in short videos, 10 studies investigated the conditions or mechanisms under which these persuasive effects occur. These studies provided evidence that the persuasive impact of health messages in short videos was moderated by individuals’ sociodemographic factors (eg, age, race, and therapeutic background [16,35]), health and media literacy [33,40], political ideology and beliefs [28], message dissemination phases [31], and congruence between exposed and evaluated cases [30]. Generally, narrative techniques were found to be particularly beneficial for individuals with low literacy because of their limited self-efficacy in understanding and processing information [33,40]. In addition, health messages in short videos were particularly effective for those initially skeptical about the topic, as others might have already been receptive to the issue [28,35].

A mere 3 studies within the synthesis explored the mediating effect, leaving the mechanism by which the design of health messages in short videos influences people’s attitudes and behaviors unclear. Ewing et al [36] found that avoidance parenting mediated the relationship between short videos instructing parents on assisting children in coping with fears and the children’s subsequent behaviors. González-Cabrera and Igartua [40] and Violeau et al [37] found that engagement with narratives and characters mediated the persuasive efficacy of short video health messages. These findings potentially suggest that, to bolster persuasive outcomes, relevant stakeholders might consider strategizing short video designs that enhance viewer engagement and consequently persuasive results. However, it is important to exercise caution concerning the context, as such engagement with narratives and characters might be associated with emotional arousal and could be less preferable than presenting factual matters, which aids individuals in coping with uncertainty during periods of heightened crisis severity [51].

## Discussion

### Principal Findings

Given the increasing popularity of short video platforms, discerning how to capitalize on short videos for disseminating health information has emerged as a pressing concern for health researchers and practitioners [13]. Although previous systematic reviews have primarily concentrated on the impact of health communication in short videos on user engagement [17], this study presents a systematic review of existing research exploring the persuasive outcomes of health communication in short videos, which is crucial for guiding the effective design of health messages and campaigns.

A literature search yielded 18 papers, suggesting that this area remains relatively underexplored. This synthesis unveils that the design of health messages in short videos encompasses (1) topic relevance, (2) topic themes, and (3) narrative and video appeal techniques. The persuasive effects of the design of health messages were largely mixed, with evidence supporting and contradicting the effects within each category. In the following sections, we delve deeper into the insights obtained from this review and offer guidance for future research.

### Addressing Methodological Limitations: Defining Short Videos and Expanding Sample Diversity and Research Design

This synthesis highlights the fact that extant studies lack a consistent definition of short videos. Remarkably, this issue is not solely present in the examined studies, but also in the broader research on short videos. For example, Cao et al [52] defined short videos as those lasting less than 1 minute, whereas Kaye et al [53] defined short videos as those shorter than 5 minutes. Owing to the vague definitions, the lengths of short videos in the analyzed studies vary, ranging from 25 seconds to 20 minutes. When the short video platform TikTok was launched in 2016, it had a time-limit restriction of 15 seconds [54]. Subsequently, TikTok extended its maximum video length to 1 minute, 3 minutes, and 10 minutes. In light of these continuous adjustments, it is debatable whether the video length alone is adequate for defining short videos. If videos up to 10 minutes are still considered short videos, how are they differentiated from general videos, such as those uploaded to YouTube? A potential solution involves considering other salient characteristics of short videos, such as low-cost production. For instance, short video platforms equip users with features such as music, animation, and visual effect editing, allowing them to effortlessly produce videos and blur the lines between producers and consumers [9,53]. Further discussion regarding alternative features beyond the time limit for defining short videos is warranted.

In addition, the samples in the examined studies display biases in terms of location and education. This could potentially be attributed to the review’s inclusion criteria, which only considered studies written in English, possibly overlooking research from diverse locations not written in English. Notwithstanding this limitation, most of the investigated studies in this review were conducted in Western countries, with only 4 studies undertaken in Africa and Asia. The Asia Pacific region, in fact, boasts a greater number of short video platform users than other regions [55]. Countries such as Indonesia, Brazil, and Mexico also exhibit a high number of users accessing short video platforms [56]. More importantly, previous research has shown that individuals from different countries exhibit diverse reactions to health information. For example, Koreans and Hong Kongers demonstrated higher trust in experience-based health information compared with Americans [57]. Hong Kongers and Americans also displayed distinct heuristic and cognitive mechanisms for processing persuasive health messages [58]. Consequently, the generalizability of the persuasive effects of health messages in short videos, as observed in the West, to other countries remains uncertain. Therefore, we advocate for future research to investigate health messages in short videos in non-Western contexts or to conduct cross-cultural studies comparing persuasive effects across different countries.

The education level of study participants is also disproportionately represented. The majority of participants in the analyzed studies possessed at least some college education. However, previous research indicates that individuals with lower educational levels face higher risks of problematic smartphone use [59] and are more prone to develop internet addiction [60]. Moreover, this group exhibits less trust in most health information sources, such as the internet, magazines, and newspapers [61], and is more susceptible to health misinformation [62]. This suggests that people with lower educational levels might be more inclined to overuse short video platforms and they may process health information differently than those with higher educational levels. Consequently, investigating the persuasive effect of health messages in short videos among individuals with lower educational levels could yield additional insights into the topic.

In addition, the literature examined in this review uses limited methods, with approximately all studies exclusively relying on web-based or laboratory experiments. This limitation in methods may hinder a comprehensive understanding of the persuasive effect of health communication in short videos. To enrich the understanding, future research can use various methods, incorporating interviews, surveys, experience sampling, and even mixed methods. In addition, only a few studies within the synthesis measured the actual behaviors. Although perceptions, beliefs, and attitudes are crucial antecedents of behaviors [47], inconsistencies between attitudes and behaviors may arise [49]. To address this gap, researchers should consider using field experiments that measure participants’ behaviors both before and after the study. For example, following the approach of Qiu and Kumar [63], investigators could create fictitious accounts on short video platforms and require participants in experimental and control groups to follow different accounts through which health messages with distinct designs are disseminated. In doing so, researchers can more accurately assess people’s behaviors and reactions to the design of health messages in short videos.

### Navigating Design and Theory: Exploring Content, Narrative Techniques, and Theoretical Mechanisms

Overall, when comparing various topics, themes, and narrative techniques, researchers found that identifying relevant topics and appropriate themes was effective in persuasive outcomes for health communication in short videos. However, the persuasive effects of different narrative techniques remain uncertain, possibly because of the abbreviated length of the short videos and viewers’ limited exposure. In addition, the persuasive impact of the content is dependent on the sample. Consistent with previous research demonstrating that interventions promoting attitude change have the largest effect size among individuals with poorer baseline attitudes and no prior training on the focal issue [64], the examined studies suggest that participants’ baseline attitudes and prior training may potentially influence the persuasive effects of health communication in short videos. This underscores the fact that the content for health communication in short videos may be particularly beneficial for those with limited knowledge and unfavorable attitudes.

Furthermore, the effectiveness of narrative techniques for health communication in short videos remains ambiguous. Several studies found that the anticipated superior narrative style did not outperform alternative methods [65,66], aligning with the findings in the study by Tukachinsky [49] that narrative techniques used in videos had a smaller effect size than textual and video game stimuli, possibly because of reduced mental simulation requirements [50]. Consequently, there is an urgent need for further research to examine the overall effectiveness of narrative techniques for health communication in short videos and the boundary conditions of such narrative techniques, such as the context of the disease [31] and target audience [40].

However, it is encouraging to note that numerous studies in this review have explored moderators, which shed light on the intricate connection between the design of health-related short videos and their intended viewers. For instance, when designing health-related short videos, it is crucial for practitioners to focus on audiences who might initially be resistant to the health message, as others might have already accepted the messages. For example, when promoting vaccination, special attention could be directed toward individuals with a mistrust of governmental entities, as they may be less likely to heed governmental health advisories [28]. The chosen narrative techniques should be tailored to the audience. For audiences with limited media and health literacy, practitioners might opt for techniques that spark their emotional responses, such as firsthand testimonials or compelling storylines.

Although we have gleaned some understanding about moderators, the mechanisms underlying the persuasive effects of health messages in short videos have yielded mixed results and remain underexplored. Only a few studies in the synthesis have examined the mediation effect, leaving the mechanism in which the design of health messages in short videos influences people’s attitudes and behaviors unclear. Hence, we encourage future research to investigate the mechanisms underlying the observed effects, with a particular emphasis on two aspects: (1) individuals’ emotional reactions and (2) their cognitive processing. This is because Updegraff and Rothman [67] posited that persuasive health messages activate individuals’ emotional responses, such as fear, happiness, and sadness, which in turn guide their behaviors. In addition, the elaboration likelihood model suggests that message attributes influence the attention and consideration individuals devote to messages, subsequently affecting their susceptibility to persuasion [68]. In this context, individuals’ emotional reactions and cognitive processing of health messages in short videos may affect the persuasive effects of messages, highlighting the need for researchers to further examine these variables as mediators.

In addition, although most of the studies in the synthesis are theoretically grounded, the theories and concepts used have been widely examined, particularly in persuasion research. Few studies have explored how the unique aspects of short videos extend or challenge traditional persuasion theories. Given the distinct features of short videos, researchers can endeavor to expand existing theories or develop new ones to comprehend the persuasive effects of short videos. For example, owing to their brief duration, attention-grabbing features are vital for short video producers. However, attention-grabbing elements, such as humor, can act as a double-edged sword in persuasion, as they might compromise the credibility of health care messages [69] and diminish risk perception [31]. Thus, striking a delicate balance between garnering attention and persuading through short videos may present a challenge for health practitioners. Further theoretical discussion and insights are required.

### Limitations

This review has several limitations. First, the initial keywords used for searching might have missed studies if they did not mention the search strings in their title, abstract, or keywords. For instance, while encompassing a broad array of short video platforms, this review may omit platforms that are not specified in the search string. To enhance comprehensiveness, future research should consider expanding the search strings to capture a wider range of short video platforms. Second, the purpose of this review is to provide a comprehensive overview of existing studies and offer guidance for researchers and practitioners, which led to the adoption of a fine-grained systematic review approach rather than a meta-analysis. The diverse research focuses among the included articles also impede the feasibility of conducting a meta-analysis. Nevertheless, as the number of studies in this area continues to grow, a more advanced meta-analysis may become necessary in the future. Finally, given the limited number of studies included in this review, our findings regarding persuasive impacts might be inconclusive. As more research surfaces in the future, a clear understanding of persuasion effects may be established.

## Appendix

Multimedia Appendix 1 PRISMA (Preferred Reporting Items for Systematic Reviews and Meta-Analyses) checklist.

Multimedia Appendix 2 Search strategies for all searched databases.

Multimedia Appendix 3 Raw data set (4118 papers).

Multimedia Appendix 4 Removed duplicates (3810 papers).

Multimedia Appendix 5 Screened by title and abstract (36 papers).

Multimedia Appendix 6 Final data set (18 papers).

Multimedia Appendix 7 Full coding.

## Abbreviations

PRISMA: Preferred Reporting Items for Systematic Reviews and Meta-Analyses
RQ: research question
WTS: waterpipe tobacco smoking
