# Supplementary material for: Examining the Persuasive Effects of Health Communication in Short Videos: Systematic Review
Source: J Med Internet Res. 2023 Oct 13;25:e48508. doi: 10.2196/48508 (PMC10612001; doi:10.2196/48508)
Supplement: Multimedia Appendix 2 [file jmir_v25i1e48508_app2.pdf]

Below, we provide the search strings employed for each database:

## ACM

(Abstract:(("short video" OR tiktok OR douyin OR "youtube shorts" OR "instagram reels" OR triller OR "snapchat spotlight" OR vine OR "facebook shorts")

AND (persua* OR impact OR effect OR outcome OR belief OR attitude OR behavior OR behaviour OR intention OR knowledge)

AND (health OR medic* OR clinical OR disease OR disabilit* OR disorder OR ill* OR well-being OR wellbeing)))

OR

(Title:(("short video" OR tiktok OR douyin OR "youtube shorts" OR "instagram reels" OR triller OR "snapchat spotlight" OR vine OR "facebook shorts")

AND (persua* OR impact OR effect OR outcome OR belief OR attitude OR behavior OR behaviour OR intention OR knowledge)

AND (health OR medic* OR clinical OR disease OR disabilit* OR disorder OR ill* OR well-being OR wellbeing)))

OR

(Keyword:(("short video" OR tiktok OR douyin OR "youtube shorts" OR "instagram reels" OR triller OR "snapchat spotlight" OR vine OR "facebook shorts")

AND (persua* OR impact OR effect OR outcome OR belief OR attitude OR behavior OR behaviour OR intention OR knowledge)

AND (health OR medic* OR clinical OR disease OR disabilit* OR disorder OR ill* OR well-being OR wellbeing)))

Apply filters: research article

## PsycINFO

(("short video" OR tiktok OR douyin OR "youtube shorts" OR "instagram reels" OR triller OR "snapchat spotlight" OR vine OR "facebook shorts")

AND (persua* OR impact OR effect OR outcome OR belief OR attitude OR behavior OR behaviour OR intention OR knowledge)

AND (health OR medic* OR clinical OR disease OR disabilit* OR disorder OR ill* OR well-being OR wellbeing)).ab

OR

(("short video" OR tiktok OR douyin OR "youtube shorts" OR "instagram reels" OR triller OR "snapchat spotlight" OR vine OR "facebook shorts")

AND (persua* OR impact OR effect OR outcome OR belief OR attitude OR behavior OR behaviour OR intention OR knowledge)

AND (health OR medic* OR clinical OR disease OR disabilit* OR disorder OR ill* OR well-being OR wellbeing)).ti

OR

(("short video" OR tiktok OR douyin OR "youtube shorts" OR "instagram reels" OR triller OR "snapchat spotlight" OR vine OR "facebook shorts")

AND (persua* OR impact OR effect OR outcome OR belief OR attitude OR behavior OR behaviour OR intention OR knowledge)

AND (health OR medic* OR clinical OR disease OR disabilit* OR disorder OR ill* OR well-being OR wellbeing)).id

## Web of Science

TI=(("short video" OR tiktok OR douyin OR "youtube shorts" OR "instagram reels" OR triller OR "snapchat spotlight" OR vine OR "facebook shorts")

AND (persua* OR impact OR effect OR outcome OR belief OR attitude OR behavior OR behaviour OR intention OR knowledge)

AND (health OR medic* OR clinical OR disease OR disabilit* OR disorder OR ill* OR well-being OR wellbeing))

OR

AB=(("short video" OR tiktok OR douyin OR "youtube shorts" OR "instagram reels" OR triller OR "snapchat spotlight" OR vine OR "facebook shorts")

AND (persua* OR impact OR effect OR outcome OR belief OR attitude OR behavior OR behaviour OR intention OR knowledge)

AND (health OR medic* OR clinical OR disease OR disabilit* OR disorder OR ill* OR well-being OR wellbeing))

OR

AK=(("short video" OR tiktok OR douyin OR "youtube shorts" OR "instagram reels" OR triller OR "snapchat spotlight" OR vine OR "facebook shorts")

AND (persua* OR impact OR effect OR outcome OR belief OR attitude OR behavior OR behaviour OR intention OR knowledge)

AND (health OR medic* OR clinical OR disease OR disabilit* OR disorder OR ill* OR well-being OR wellbeing))

Document types: article or proceeding papers

## Communication & Mass Media Complete

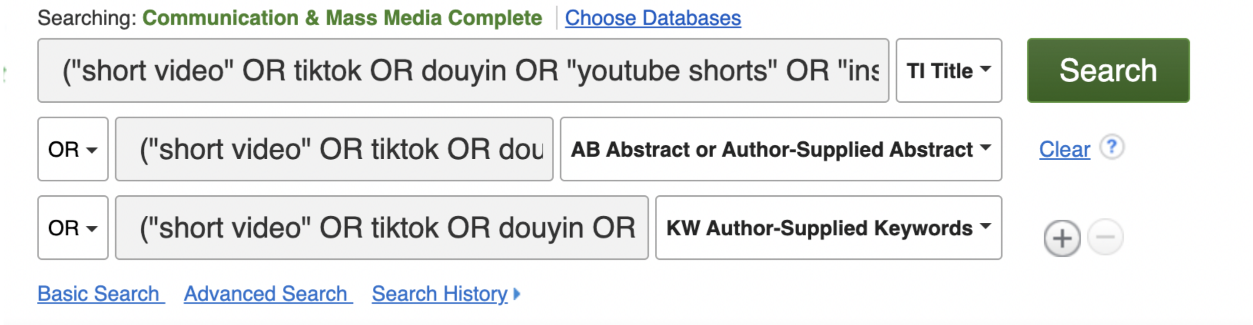


Each input field contains:
("short video" OR tiktok OR douyin OR "youtube shorts" OR "instagram reels" OR triller OR "snapchat spotlight" OR vine OR "facebook shorts")

AND (persua* OR impact OR effect OR outcome OR belief OR attitude OR behavior OR behaviour OR intention OR knowledge)

AND (health OR medic* OR clinical OR disease OR disabilit* OR disorder OR ill* OR well-being OR wellbeing)

## MEDLINE

(("short video" OR tiktok OR douyin OR "youtube shorts" OR "instagram reels" OR triller OR "snapchat spotlight" OR vine OR "facebook shorts")

AND (persua* OR impact OR effect OR outcome OR belief OR attitude OR behavior OR behaviour OR intention OR knowledge)

AND (health OR medic* OR clinical OR disease OR disabilit* OR disorder OR ill* OR well-being OR wellbeing)).ab

OR

(("short video" OR tiktok OR douyin OR "youtube shorts" OR "instagram reels" OR triller OR "snapchat spotlight" OR vine OR "facebook shorts")

AND (persua* OR impact OR effect OR outcome OR belief OR attitude OR behavior OR behaviour OR intention OR knowledge)

AND (health OR medic* OR clinical OR disease OR disabilit* OR disorder OR ill* OR well-being OR wellbeing)).ti

OR

(("short video" OR tiktok OR douyin OR "youtube shorts" OR "instagram reels" OR triller OR "snapchat spotlight" OR vine OR "facebook shorts")

AND (persua* OR impact OR effect OR outcome OR belief OR attitude OR behavior OR behaviour OR intention OR knowledge)

AND (health OR medic* OR clinical OR disease OR disabilit* OR disorder OR ill* OR well-being OR wellbeing)).id

## EMBASE

(("short video" OR tiktok OR douyin OR "youtube shorts" OR "instagram reels" OR triller OR "snapchat spotlight" OR vine OR "facebook shorts")

AND (persua* OR impact OR effect OR outcome OR belief OR attitude OR behavior OR behaviour OR intention OR knowledge)

AND (health OR medic* OR clinical OR disease OR disabilit* OR disorder OR ill* OR well-being OR wellbeing)):ti,ab,kw AND ('article'/it OR 'conference paper'/it)

## CINAHL

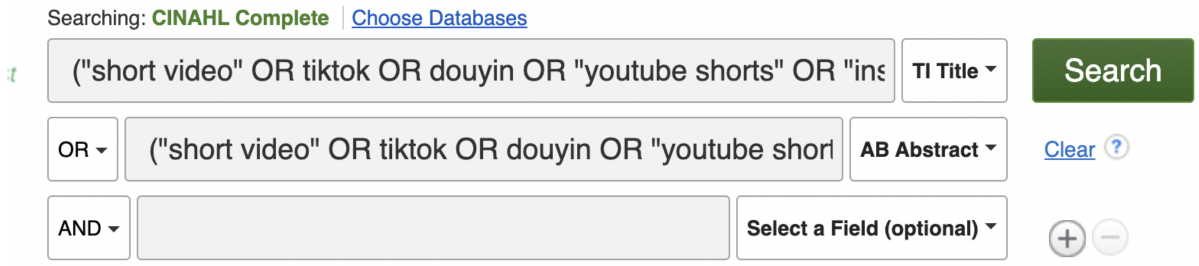


Each input field contains: ("short video" OR tiktok OR douyin OR "youtube shorts" OR "instagram reels" OR triller OR "snapchat spotlight" OR vine OR "facebook shorts")

AND (persua* OR impact OR effect OR outcome OR belief OR attitude OR behavior OR behaviour OR intention OR knowledge)

AND (health OR medic* OR clinical OR disease OR disabilit* OR disorder OR ill* OR well-being OR wellbeing)

Limiters: research article

## Scopus

( ABS ( ( "short video" OR tiktok OR douyin OR "youtube shorts" OR "instagram reels" OR triller OR "snapchat spotlight" OR vine OR "facebook shorts" ) AND ( persua* OR impact OR effect OR outcome OR belief OR attitude OR behavior OR behaviour OR intention OR knowledge ) AND ( health OR medic* OR clinical OR disease OR disabilit* OR disorder OR ill* OR well-being OR wellbeing ) ) ) OR ( TITLE ( ( "short video" OR tiktok OR douyin OR "youtube shorts" OR "instagram reels" OR triller OR "snapchat spotlight" OR vine OR "facebook shorts" ) AND ( persua* OR impact OR effect OR outcome OR belief OR attitude OR behavior OR behaviour OR intention OR knowledge ) AND ( health OR medic* OR clinical OR disease OR disabilit* OR disorder OR ill* OR well-being OR wellbeing ) ) ) OR ( KEY ( ( "short video" OR tiktok OR douyin OR "youtube shorts" OR "instagram reels" OR triller OR "snapchat spotlight" OR vine OR "facebook shorts" ) AND ( persua* OR impact OR effect OR outcome OR belief OR attitude OR behavior OR behaviour OR intention OR knowledge ) AND ( health OR medic* OR clinical OR disease OR disabilit* OR disorder OR ill* OR well-being OR wellbeing ) ) ) AND ( LIMIT-TO ( DOCTYPE , "ar" ) OR LIMIT-TO ( DOCTYPE , "cp" ) )

## Google Scholar

("short video" OR tiktok OR douyin OR "youtube shorts" OR "instagram reels" OR triller OR "snapchat spotlight" OR vine OR "facebook shorts")

AND (persua* OR impact OR effect OR outcome OR belief OR attitude OR behavior OR behaviour OR intention OR knowledge)

AND (health OR medic* OR clinical OR disease OR disabilit* OR disorder OR ill* OR well-being OR wellbeing)

*We reviewed the first 10 pages to determine if any relevant articles were missing.

## ProQuest Dissertation & Theses Global

abstract(("short video" OR tiktok OR douyin OR "youtube shorts" OR "instagram reels" OR triller OR "snapchat spotlight" OR vine OR "facebook shorts")

AND (persua* OR impact OR effect OR outcome OR belief OR attitude OR behavior OR behaviour OR intention OR knowledge)

AND (health OR medic* OR clinical OR disease OR disabilit* OR disorder OR ill* OR well-being OR wellbeing)) OR title(("short video" OR tiktok OR douyin OR "youtube shorts" OR "instagram reels" OR triller OR "snapchat spotlight" OR vine OR "facebook shorts")

AND (persua* OR impact OR effect OR outcome OR belief OR attitude OR behavior OR behaviour OR intention OR knowledge)

AND (health OR medic* OR clinical OR disease OR disabilit* OR disorder OR ill* OR well-being OR wellbeing)) OR diskw(("short video" OR tiktok OR douyin OR "youtube shorts" OR "instagram reels" OR triller OR "snapchat spotlight" OR vine OR "facebook shorts")

AND (persua* OR impact OR effect OR outcome OR belief OR attitude OR behavior OR behaviour OR intention OR knowledge)

AND (health OR medic* OR clinical OR disease OR disabilit* OR disorder OR ill* OR well-being OR wellbeing))

After including search results, we proceed through the steps outlined in Figure 1: PRISMA diagram showing the flow of studies.
